# Impact of Lactate on 30-Day All-Cause Mortality in Patients with and without Out-of-Hospital Cardiac Arrest Due to Cardiogenic Shock

**DOI:** 10.3390/jcm11247295

**Published:** 2022-12-08

**Authors:** Jonas Rusnak, Tobias Schupp, Kathrin Weidner, Marinela Ruka, Sascha Egner-Walter, Jan Forner, Thomas Bertsch, Maximilian Kittel, Kambis Mashayekhi, Péter Tajti, Mohamed Ayoub, Michael Behnes, Ibrahim Akin

**Affiliations:** 1 Department of Cardiology, Angiology, Haemostaseology and Medical Intensive Care, University Medical Centre Mannheim, Medical Faculty Mannheim, Heidelberg University, 68167 Mannheim, Germany; 2 European Center for AngioScience (ECAS), German Center for Cardiovascular Research (DZHK) Partner Site Heidelberg/Mannheim, 68167 Mannheim, Germany; 3 Institute of Clinical Chemistry, Laboratory Medicine and Transfusion Medicine, Nuremberg General Hospital, Paracelsus Medical University, 90419 Nuremberg, Germany; 4 Institute for Clinical Chemistry, Faculty of Medicine Mannheim, Heidelberg University, 68167 Mannheim, Germany; 5 Department of Internal Medicine and Cardiology, Mediclin Heart Centre Lahr, 77933 Lahr, Germany; 6 Gottsegen György National Cardiovascular Center, 1096 Budapest, Hungary; 7 Division of Cardiology and Angiology, Heart Center University of Bochum, 32545 Bad Oeynhausen, Germany

**Keywords:** cardiogenic shock, lactate, prognosis, mortality, OHCA

## Abstract

In patients with cardiogenic shock (CS) due to myocardial infarction, elevated lactate levels are known to be negative predictors. Studies regarding the prognostic impact in patients with CS complicated by out-of-hospital cardiac arrest (OHCA) are limited. Two hundred and sixty-three consecutive patients with CS were included. The prognostic value of lactate on days 1, 2, 3, 4 and 8 was tested stratified by OHCA and non-OHCA. Statistical analyses included the univariable *t*-test, Spearman’s correlation, C-statistics, Kaplan–Meier analyses, as well as multivariable mixed analysis of variance (ANOVA) and Cox proportional regression analyses. The primary endpoint of all-cause mortality occurred in 49.4% of the non-OHCA group and in 63.4% of the OHCA group. Multivariable regression models showed an association of lactate values with 30-day all-cause mortality in the non-OHCA (*p* = 0.024) and OHCA groups (*p* = 0.001). In Kaplan–Meier analyses, patients with lactate levels ≥ 4 mmol/L (log-rank *p* = 0.001) showed the highest risk for 30-day all-cause mortality in the non-OHCA as well as in the OHCA group. However, in C-statistics lactate on days 1 and 8 had a better discrimination for 30-day all-cause mortality in the OHCA group compared to the non-OHCA group. In conclusion, patients presenting with CS lactate levels showed a good prognostic performance, with and without OHCA. Especially, lactate levels on days 1 and 8 were more accurate in the discrimination for all-cause mortality in CS-patients with OHCA.

## 1. Introduction

Besides the positive impact of early percutaneous coronary intervention (PCI) on the survival of patients with acute myocardial infarction (AMI) complicated by cardiogenic shock (CS), no significant mortality-reducing intervention or treatment is known so far [1,2]. Therefore, mortality rates remain high in patients with CS, regardless of its cause. Extracorporeal life support (ECLS) might be an option to reduce mortality if selected for the right patients [3]. However, selecting the right patient and timing are crucial as well as challenging. Differentiation of the stages of CS is a helpful approach [4]. However, to identify patients with deteriorating CS more clearly, biomarkers or clinical parameters are needed. Lactate is a biomarker that has been shown to have a negative predictive impact in critically ill patients, patients with shock as well as CS, and is part of the risk scores specified for CS [5,6,7,8,9,10,11,12,13]. Furthermore, several retrospective and prospective observational studies and registries showed that elevated lactate values were predictive of 30-day all-cause mortality [14,15,16]. Most recently, Jentzer et al. postulated that in patients with CS, severe acidosis should be used as a marker for refractory or hemometabolic shock [17,18]. For this matter, lactate levels should be considered [17,18]. 

In patients with cardiac arrest, especially out-of-hospital cardiac arrest (OHCA), the prognostic value of lactate has been examined [19,20,21,22,23,24]. Cardiac arrest in patients with CS is a feared complication that aggravates the severity of shock, and survival rates remain low despite improvements in treatment and the implementation of ECLS [1,3,4,25,26,27]. Furthermore, in a retrospective analysis of 9898 patients admitted to intensive care unit (ICU) with shock, cardiac arrest and the underlying rhythm both influenced hospital mortality and the severity of shock [28]. Therefore, a more distinct investigation of the special patient cohort with CS and OHCA is needed. 

However, most studies focusing on OHCA have consisted of patients with sepsis or included all patients with cardiac arrest irrespective of the definite cause. Data regarding the different prognostic impact of lactate in the specific cohort of CS-patients with or without OHCA is limited. Therefore, the present study investigates the prognostic impact of lactate levels on different timepoints in patients with CS and the subgroup of OHCA.

## 2. Materials and Methods

### 2.1. Patient Study, Design, and Data Collection

The present study prospectively included all consecutive patients presenting with CS of all entities on admission to the internal ICU at the University Medical Center Mannheim, Germany, from June 2019 to May 2021. All relevant clinical data related to the index event were documented using the electronic hospital information system as well as the IntelliSpace Critical Care and Anesthesia information system (ICCA, Philips, Philips GmbH Market DACH, Hamburg, Germany) implemented on the ICU, organizing patient data, including admission documents, vital signs, laboratory values, treatment data and consult notes. 

The presence of CS, as well as important laboratory data, ICU-related scores, hemodynamic measurements and ventilation parameters were assessed on the day of admission. In order to provide a more thorough understanding of the changes in these parameters, particularly during the first week of the ICU treatment, these parameters were again evaluated on days 2, 3, 4 and 8. Changes in lactate, especially in the first days, are more precise than static measurements alone [29]. 

Further data being documented contained baseline characteristics, prior medical history, length index hospital stay, data derived from imaging diagnostics as well as pharmacological therapies. Documentation of source data was performed by intensivists and ICU nurses during routine clinical care. 

The present study derived from an analysis of the “Cardiogenic Shock Registry Mannheim” (CARESMA), representing a prospective single-center registry including consecutive patients presenting with CS being acutely admitted to the ICU for internal medicine of the University Medical Center Mannheim (UMM), Germany (clinicaltrials.gov identifier: NCT05575856). The registry was carried out according to the principles of the Declaration of Helsinki and was approved by the medical ethics committee II of the Medical Faculty Mannheim, University of Heidelberg, Germany.

### 2.2. Inclusion and Exclusion Criteria, Study Endpoints

For the present study, all consecutive patients with CS of all causes and lactate values on admission were included. Ten out of a total of two hundred and seventy-three patients with CS had missing lactate values at the time of admission. Therefore, the final study cohort comprised 263 patients. Risk stratification was performed according to lactate levels documented on the day of admission (i.e., day 1). Patients without documented lactate on admission or patients with OHCA not regaining ROSC before admission to ICU were excluded from the present study. No further exclusion criteria were applied. 

Diagnosis of CS was determined according to the current recommendations of the Acute Cardiovascular Care Association of the European Society of Cardiology [30]. Accordingly, CS was defined by hypotension (SBP < 90 mmHg) for more than 30 min despite adequate filling status or need for vasopressor or inotropic therapy to achieve SBP > 90 mmHg. Additionally, signs for end-organ hypoperfusion must be present such as oliguria with urine output < 30 mL/hour, altered mental status, cold clammy skin and increased lactate > 2 mmol/L. Causes of CS were not limited, and comprised AMI, arrythmias, acute decompensated heart failure, pulmonary embolism, valvular defect, cardiomyopathy and aortic dissection. 

The primary endpoint of the study was all-cause mortality at 30 days, which was documented using the electronic hospital information system and by directly contacting state resident registration offices (‘bureau of mortality statistics’). Identification of patients was verified by name, surname, date of birth and registered living address. No patient was lost to follow-up with regard to all-cause mortality at 30 days. 

### 2.3. Measurement of Plasma Lactate

Lactate values were derived directly on admission to ICU. Therefore, arterial blood samples were taken in specific blood gas syringes (safePICO, Radiometer GmbH, Krefeld, Germany). Then, the blood samples were analyzed in two blood gas analyzers (ABL 825, Radiometer Medical ApS, Brønshøj, Denmark), which were on site at the ICU. 

### 2.4. Statistical Methods

Quantitative data are presented as mean ± standard error of mean (SEM), median and interquartile range (IQR) and ranges depending on the distribution of the data. They were compared using the Student’s *t*-test for normally distributed data or the Mann–Whitney U test for nonparametric data. Deviations from a Gaussian distribution were tested by the Kolmogorov–Smirnov test. Qualitative data are presented as absolute and relative frequencies and were compared using the Chi-square test or the Fisher’s exact test, as appropriate. Box plots for lactate levels were created for the comparisons of survivors and non-survivors for the days 1, 2, 3, 4 and 8. Spearman’s rank correlation for nonparametric data was used to test the association of lactate levels with medical and laboratory parameters on day 1.

Using a one-factorial repeated measures ANOVA, the lactate levels of days 1, 2, 3 and 4 were separately analyzed for survivors and non-survivors in order to assess the effect of time on biomarker levels. The sphericity was tested for by using Mauchly’s test and a Huyn–Feldt correction was applied to the one-factorial repeated measures ANOVA results in case of not fulfilling the spherical assumption. The Huyn–Feldt correction was preferred over the Greenhouse–Geisser correction, for the latter tends to increase the risk for type II error by being more conservative [31].

Kaplan–Meier analyses according to lactate levels on admission were performed within the two groups of non-OHCA and OHCA. Univariable hazard ratios (HR) were given together with 95% confidence intervals. Thereafter, multivariable Cox regression models were developed using the “forward selection” option, where only statistically significant variables (*p* < 0.05) were included and analyzed simultaneously. 

Results of all statistical tests were considered significant for *p* ≤ 0.05. SPSS (Version 25, IBM, Armonk, New York, NY, USA) and GraphPad Prism (Version 9, GraphPad Software, San Diego, CA, USA) were used for statistics. 

## 3. Results

### 3.1. Study Population

As presented in Table 1, baseline characteristics differed in some parts between the two groups. Patients with OHCA were younger (66 years vs. 77 years) and more often male (73.3% vs. 51.9%; *p* = 0.001). Regarding physiological parameters measured on admission, patients with OHCA had a lower body temperature (35.2 °C vs. 36.2 °C; *p* = 0.001) and a lower respiratory rate (19/min vs. 20/min; *p* = 0.011). Furthermore, patients with OHCA showed a higher systolic blood pressure (114 mmHg vs. 105 mmHg; *p* = 0.009). However, these results might be due to the administration of inotropes and vasopressors. Lower rates of cardiovascular risk factors such as arterial hypertension 63.4% vs. 79.6%; *p* = 0.004), diabetes mellitus (28.0% vs. 46.3%; *p* = 0.003) and hyperlipidemia (39.6% vs. 59.3%; *p* = 0.002) were present in the group of OHCA. In line with this, patients with OHCA were less likely to suffer from congestive heart failure (17.8% vs. 46.3%; *p* = 0.001), atrial fibrillation (19.8% vs. 40.1 5; *p* = 0.001), chronic kidney disease (15.8% vs. 45.1%; *p* = 0.001) and stroke (5.0% vs. 19.1%; *p* = 0.001). Moreover, lower rates of the intake of beta blocker (37.9% vs. 61.1%; *p* = 0.001), diuretics (29.5% vs. 54.3%; *p* = 0.001), acetylsalicylic acid (17.8% vs. 35.8%; *p* = 0.002) and statin (31.7% vs. 52.4%; *p* = 0.009) were seen in patients with OHCA. 

On admission, echocardiographic parameters did not differ significantly between the groups except of TAPSE, which was higher in patients with OHCA (17 mm vs. 14 mm; *p* = 0.015) (Table 2). Due to the specific group assignment, the classification of CS and the site of cardiac arrest (IHCA vs. OHCA) were different between the groups. Furthermore, patients with OHCA were more likely to suffer from AMI (57.4% vs. 42.6%), whereas patients without OHCA had higher rates of acute decompensated heart failure (ADHF; 15.8% vs. 30.2%). Regarding cardiac arrest, more shockable rhythms (61.0% vs. 6.8%; *p* = 0.001) and a longer time to return of spontaneous circulation (ROSC; 15 min vs. 10 min; *p* = 0.028) were seen in patients with OHCA. Mechanical ventilation (95.1% vs. 34.8%; *p* = 0.001) with a longer duration of ventilation (5 days vs. 1 day; *p* = 0.001) and a higher PaO_2_ on admission (112 mmHg vs. 98 mmHg; *p* = 0.047) as well as higher dosages of norepinephrine (0.2 µg/kg/min vs. 0.1 µg/kg/min; *p* = 0.001) were more often needed in patients with OHCA. Patients with OHCA showed a lower pH value (7.25 vs. 7.31; *p* = 0.001) with higher values of lactate (3.8 mmol/L vs. 2.8 mmol/L; *p* = 0.021). The levels of serum potassium (4.2 mmol/L vs. 4.4 mmol/L; *p* = 0.004), creatinine (1.41 mg/dL vs. 1.53 mg/dL; *p* = 0.037), NTproBNP (1047 pg/mL vs. 7944 pg/mL; *p* = 0.001) and CRP (4 mg/L vs. 25 mg/L; *p* = 0.001) were lower in OHCA patients, whereas the values of hemoglobin (13.2 g/dL vs. 11.9 g/dL; *p* = 0.001), white blood cells (16.39 × 10^6^/mL vs. 13.31 × 10^6^/mL, *p* = 0.001), D-dimer (24.18 mg/L vs. 2.86 mg/L; *p* = 0.001), aspartate aminotransferase (AST; 167 U/L vs. 94 U/L; *p* = 0.005) and alanine aminotransferase (ALT; 115 U/L vs. 51 U/L; *p* = 0.001) were higher.

Regarding outcome parameters, the primary endpoint of 30-day all-cause mortality was significantly higher in patients with OHCA (63.4% vs. 49.4%; *p* = 0.027) as well as time on ICU (6 days vs. 3 days; *p* = 0.001) and death on ICU (63.4% vs. 48.8%; *p* = 0.021).

### 3.2. Association with Clinical and Laboratory Data

Table 3 outlines the correlation of lactate levels with clinical and laboratory parameters in the groups of non-OHCA and OHCA patients. In both groups, lactate inversely correlated with platelet count (r = −0.174; *p* = 0.034 and r = −0.289; *p* = 0.005), albumin levels (r = −0.177; *p* = 0.040 and r = −0.465; *p* = 0.001) and intensive care days (r = −0.261; *p* = 0.001 and r = −0.441; *p* = 0.001), whereas significant correlations between lactate levels and PaO_2_/FiO_2_ ratio (r = −0.269; *p* = 0.011) or mechanical ventilation (r = −0.407; *p* = 0.001) were solely seen in the OHCA group. Lactate levels directly correlated with bilirubin levels (r = 0.295; *p* = 0.003 and r = 0.340; *p* = 0.012), creatine levels (r = 0.294; *p* = 0.001 and r = 0.240; *p* = 0.019), SOFA score (r = 0.296; *p* = 0.001 and r = 0.283; *p* = 0.004) and catecholamines (r = 0.258; *p* = 0.001 and r = 0.253; *p* = 0.012) in both groups. In contrast, lactate levels solely correlated directly with the APACHE II score in the non-OHCA group (r = 0.367; *p* = 0.001), whereas a positive correlation between lactate levels and procalcitonin (r = 0.551; *p* = 0.018), cardiac troponine I (cTNI; r = 0.264; *p* = 0.010) and left-ventricular ejection fraction (LVEF, r = 0.224; *p* = 0.025) was solely present in the OHCA group.

### 3.3. Prognostic Performance of Lactate Levels

**Figure 1:**
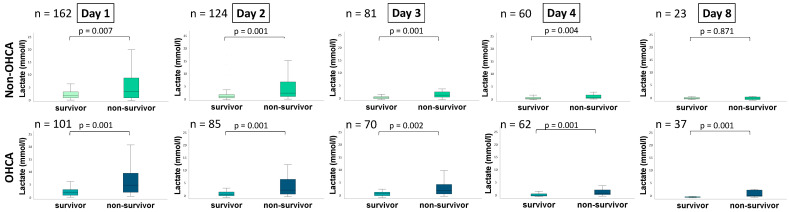
Box plots demonstrating distribution of lactate among patients with CS on days 1, 2, 3, 4 and 8 comparing 30-day survivors and non-surviors. Data is presented as median with interquartile ranges (boxes) and 5–95% percentiles (whiskers).

Box plots presenting the distribution of lactate values on days 1, 2, 3, 4 and 8 between the groups of non-OHCA and OHCA are outlined in Figure 1. Despite day 8 in the non-OHCA group, all lactate values were significantly higher in patients not surviving the hospital stay. This is in line with C-statistics revealing comparable AUCs for the discrimination of lactate levels on days 1 to 4 for 30-day all-cause mortality in both groups of non-OHCA and OHCA, as outlined in Table 4. Solely on day 8, lactate showed a poor statistical performance in the group of non-OHCA patients. Furthermore, the ROC analysis on day 1 in the non-OHCA group revealed a lower AUC for the discrimination for 30-day all-cause mortality compared to the OHCA group (0.622 vs. 0.753). On days 2 and 3, lactate levels showed a higher AUC in the non-OHCA group compared to the OHCA group, whereas on day 4 the AUC in the OHCA showed higher values.

**Figure 2:**
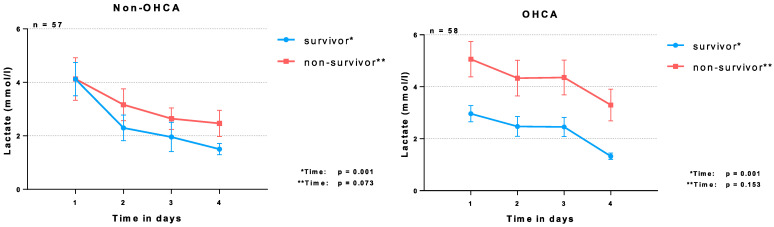
Mixed analysis of variance (ANOVA) for lactate levels within the two groups of non-OHCA and OHCA for the comparison of 30-day survivors versus non-survivors.

As illustrated in Figure 2, in the non-OHCA group time alone revealed a significant effect on the lactate levels in patients not surviving the 30-day follow-up (*p* = 0.001), whereas no effect could be seen in patients not surviving until day 30 (*p* = 0.073). This finding was similar in the OHCA group (survivors: *p*-value = 0.001; non-survivors: 0.153).

**Figure 3:**
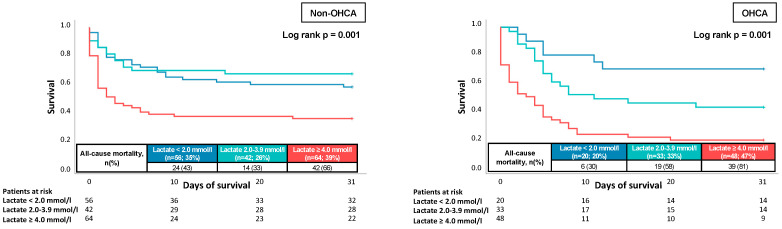
Prognostic impacts of lactate levels (lactate < 2.0 mmol/L vs. lactate 2.0–3.9 mmol/L. vs. lactate ≥ 4.0 mmol/L) on all-cause mortality at 30 days within the non-OHCA group (**left panel**), and in the OHCA group (**right panel**).

After the follow-up of 30 days, the primary endpoint of all-cause mortality occurred in 49.4% of the non-OHCA group and in 63.4% of the OHCA group. In fact, lactate levels ≥ 2 mmol/L showed an association with 30-day all-cause mortality (*p* = 0.001). However, differentiated in the Kaplan–Meier analysis (Figure 3, left panel), it could be seen that in the non-OHCA group lactate levels 2–3.9 mmol showed a nearly similar outcome compared to lactate levels < 2.0 mmol/L, whereas non-OHCA patients with lactate levels ≥ 4 mmol/L showed a clearly increased all-cause mortality at day 30 (66%; log-rank 0.001). Accordingly, the risk of 30-day all-cause mortality was highest in patients with lactate levels ≥ 4.0 mmol/L in the OHCA group (81%; log-rank *p* = 0.001). Furthermore, patients with lactate level 2.0–3.9 mmol/L showed a higher all-cause mortality after 30 days in Kaplan–Meier analysis compared to patients with <2.0 mmol/L (58%; log-rank *p* = 0.001) (Figure 3, right panel).

After multivariable adjustment, lactate on admission was associated with increased risk of all-cause mortality at 30 days in the non-OHCA (HR = 1.070; 95% CI 1.009–1.134; *p* = 0.024) and in the OHCA group (HR = 1.151; 95% CI 1.071–1.238; *p* = 0.001) (Table 5). Moreover, solely WBC (HR = 1.078; *p* = 0.001) as well as higher dosages of catecholamines (HR = 1.390; *p* = 0.007) in the group of non-OHCA) and systolic blood pressure in the OHCA group (HR = 0.991; *p* = 0.047) were associated with increased all-cause mortality at 30 days.

## 4. Discussion

The present study investigates the prognostic value of lactate in patients with CS and the differences in the subgroups of OHCA and non-OHCA. Lactate on admission was able to predict 30-day all-cause mortality in all patients with CS in multivariate Cox regression models. However, the lactate value on day 1 showed a better discrimination in patients with OHCA and lactate on day 8 was able to discriminate for 30-day all-cause mortality solely in CS-patients with OHCA. Furthermore, in Kaplan–Meier analysis the different cut-off values (2 mmol/L and 4 mmol/L) had a better prognostic differentiation in CS-patients with OHCA compared to non-OHCA.

In critically ill patients, hyperlactatemia is common in the state of severe illness [32,33,34]. Due to a combination of hypoxemia, inflammation and decreased microcirculation an increased synthesis of lactate could be seen especially in patients with shock [33,35,36]. The prognostic role of lactate has been investigated in various studies including patients with sepsis, septic shock, critical illness, CS and cardiac arrest. However, the prognostic differences of lactate at different timepoints between patients with CS complicated by OHCA and CS-patients without OHCA have not been investigated, yet.

In the present study, lactate on admission as well as in the first days of ICU treatment (days 1–8) shows a good prognostic value in patients with CS. This is in line with a sub-study of the IABP-SHOCK II trial and the corresponding registry, which demonstrated that lactate values on admission and at 8 h were good predictors of 30-day all-cause mortality in 666 patients with AMI complicated by CS [15]. Especially, lactate values after 8 h showed a good discrimination for 30-day all-casue mortality with an AUC of 0.750, which was superior to lactate on admission and lactate clearance. In the present study, lactate on admission had a similar AUC in predicting 30-day all-cause mortality in CS-patients and OHCA (AUC of 0.753), whereas in CS-patients without OHCA the AUC was lower (AUC 0.622). This indicates that the discrimination for 30-day all-cause mortality of lactate on admission might be more accurate in CS-patients with OHCA compared to patients without OHCA. However, due to the study design it cannot be differentiated whether CS-patients, especially those without OHCA, suffered from early or late onset CS. This might have an influence on the lactate value on admission. Furthermore, time to hospitalization in the OHCA group, which was beyond the scope of the study, might also have had an impact on lactate values, in particular on those assessed on admission.

Within an analysis of the CardShock registry, different quartiles of lactate values at baseline as well as after 6, 12 and 24 h were predictive of 30-day all-cause mortality in 217 consecutive CS-patients admitted to ICU [16]. These findings were consistent up to 96 h, which is in line with the present study showing that lactate values up to day 8 might discriminate for 30-day all-cause mortality. However, in the present cohort lactate values on day 8 might show a good discrimination for 30-day all-cause mortality in CS-patients with OHCA (AUC 0.863), whereas in CS-patients without OHCA C-statistics revealed a poor performance (AUC 0.479). This might be explained by the different duration of the ICU stay. The CS-patients with OHCA stayed longer on ICU compared to CS-patients with non-OCHA, indicating that the severity of CS at day 8 might be greater in the OHCA group. Therefore, lactate might have a greater prognostic impact. Furthermore, in univariate correlation lactate was associated with mechanical ventilation days solely in the cohort of CS-patients with OHCA, indicating that the duration of ventilation, which is usually longer in resuscitated patients than in patients without cardiac arrest, might have influenced the prognostic performance of lactate.

In a retrospective analysis by Jentzer et al., 1814 patients with CS were analyzed regarding the prognostic impact of lactate ≥ 5 mmol/L and a blood pH < 7.2 on 30-day all-cause mortality [18]. It could be demonstrated that either lactate ≥ 5 mmol/L or a blood pH < 7.2 were associated with an increased all-cause mortality after a follow-up of 30 days. Furthermore, lactate ≥ 5 mmol/L was able to predict all-cause mortality in patients with and without cardiac arrest, which is consistent with the findings of the present study demonstrating that a lactate value ≥ 4 mmol/L on admission shows a good prognostic performance in CS-patients with and without OHCA. However, the present study expands the knowledge by showing in the Kaplan–Meier analysis that a more precise differentiation in cut-off values ≤ 2 mmol/L and ≤ 4 mmol/L appears to solely have a prognostic benefit in CS-patients with OHCA. This finding suggests that for therapy escalation such as ECLS different approaches might appear when using lactate as an indicator for deterioration.

The underlying reasons for the increasing levels of lactate in the presence of critical illness as well as CS are various and not fully understood [37]. In acute illness, several reasons are discussed that might cause disrupted metabolism and the increased production of lactate [32,35]. Whereas lactate serves as a major renewable carbohydrate fuel and recyclable buffer in healthy humans, it accumulates in acute illness due to hypoperfusion, hypoxemia, elevated catecholamines and inflammation [37,38]. Especially, circulating catecholamines might induce lactate elevation due to an impact on skeletal muscle glycogen break down [37]. In the present study, the prognostic performance of lactate on day 1 was more precise in patients with OHCA compared to non-OHCA. This might be explained by the higher lactate levels on admission in the OHCA group and, therefore, a greater influence of lactate on outcome might result in those patients. These higher lactate values could be attributed to the fact that patients with cardiac arrest are more exposed to exogeneous and endogenous catecholamines after ROSC.

This study has several limitations. Due to the single-center and observational study design, results may be influenced by measured and unmeasured cofounding, although adjustments for potential cofounders were performed using multivariable Cox regression. Patients dying before first lactate measurements could not be included in the analysis and time to hospitalization for patients with OHCA might have influenced the results. Combining lactate with cardiac biomarkers such as troponin or Amino-terminal pro-brain- natriuretic peptide (NT-pro BNP) was not feasible due to the lower number of measurements of the cardiac biomarkers. As NT-pro BNP is a good predictor for in-hospital mortality and re-hospitalization in heart failure patients this might improve the diagnostic accuracy [39,40]. However, due to the lower number of assessments of cardiac biomarkers several patients would have been excluded and this would have biased the results. Furthermore, for lactate analysis beyond day 1 solely a small number of patients could be included due to the high mortality in the first days of CS. Lactate analysis after 6 h could not be included in the analysis due to the inconsistency of the timing of lactate measurements. Moreover, due to the study design, no conclusions can be made as to whether increases in lactate levels are caused by an increased production or lack of clearance. Finally, the effects of lactate levels on long-term outcomes were beyond the scope of the present study.

## 5. Conclusions

In conclusion, lactate on admission is a reliable tool for predicting 30-day all-cause mortality in CS-patients with or without OHCA in univariate and multivariate Cox regression models. The discrimination for 30-day all-cause mortality of lactate on admission and on day 8 was higher in CS-patients with OHCA compared to CS-patients without OHCA. Furthermore, Kaplan–Meier analysis revealed that a more precise differentiation of cut-off values beyond ≤4 mmol/L might solely have a prognostic benefit in CS-patients with OHCA and not in CS-patients without OHCA.

## Figures and Tables

**Table 1:** Baseline characteristics of patients with CS differentiated by OHCA and non-OHCA.

	All Patients (*n* = 263)		OHCA (*n* = 101)		Non-OHCA (*n* = 162)	
**Age**, median; (IQR)	73	(63–81)	66	(57–78)	77	(67–82)
**Male sex**, *n* (%)	158	(60.0)	74	(73.3)	84	(51.9)
**Body mass index**, kg/m^2^ (median, (IQR))	26.20	(24.20–30.00)	26.25	(24.23–29.88)	26.20	(24.20–30.05)
**Physiological parameters**, (median, (IQR))						
Body temperature (°C)	36.0	(34.9–36.5)	35.2	(34.0–36.2)	36.2	(35.6–36.8)
Heart rate (bpm)	88	(71–108)	84	(71–106)	90	(71–110)
Systolic blood pressure (mmHg)	109	(92–129)	114	(98–135)	105	(86–127)
Respiratory rate (breaths/min)	20	(17–24)	19	(16–22)	20	(17–25)
**Cardiovascular risk factors**, *n* (%)						
Arterial hypertension	193	(73.4)	64	(63.4)	129	(79.6)
Diabetes mellitus	103	(39.2)	28	(28.0)	75	(46.3)
Hyperlipidemia	136	(51.7)	40	(39.6)	96	(59.3)
Smoking	95	(36.1)	30	(29.7)	65	(40.1)
**Prior medical history**, *n* (%)						
Coronary artery disease	98	(37.3)	31	(30.7)	67	(41.4)
Congestive heart failure	93	(35.4)	18	(17.8)	75	(46.3)
Atrial fibrillation	85	(32.3)	20	(19.8)	65	(40.1)
Chronic kidney disease	89	(33.9)	16	(15.8)	73	(45.1)
Stroke	36	(13.7)	5	(5.0)	31	(19.1)
COPD	51	(19.4)	18	(17.8)	33	(20.4)
Liver cirrhosis	9	(3.4)	2	(2.0)	7	(4.3)
**Medication on admission**, *n* (%)						
ACE-inhibitor	92	(35.0)	29	(33.3)	63	(38.9)
ARB	46	(17.5)	12	(13.6)	34	(21.0)
Beta-blocker	132	(50.2)	33	(37.9)	99	(61.1)
ARNI	8	(3.0)	2	(2.3)	6	(3.7)
Aldosterone antagonist	40	(15.2)	13	(14.9)	27	(16.7)
Diuretics	114	(43.3)	26	(29.5)	88	(54.3)
ASA	76	(28.9)	18	(17.8)	58	(35.8)
P2Y12-inhibitor	23	(8.8)	7	(6.9)	16	(9.9)
Statin	117	(44.5)	32	(31.7)	85	(52.4)

ACE, angiotensin-converting enzyme; ARB, angiotensin receptor blockers; ARNI, angiotensin receptor neprilysin inhibitor; ASA, acetylsalicylic acid; COPD, chronic obstructive pulmonary disease; IQR, interquartile range. Level of significance *p* < 0.05.

**Table 2:** Shock-related data, follow-up data and endpoints of patients with CS differentiated by OHCA and non-OHCA.

	All Patients (*n* = 263)		OHCA (*n* = 101)		Non-OHCA (*n* = 162)		*p* Value
**Cause of CS**, *n* (%)							
Acute myocardial infarction	126	(47.9)	58	(57.4)	69	(42.6)	
Arrhythmic	31	(11.8)	12	(11.9)	19	(11.7)	
ADHF	65	(24.7)	16	(15.8)	49	(30.2)	
Pulmonary embolism	15	(5.7)	9	(8.9)	6	(3.7)	**0.003**
Valvular defect	12	(4.6)	1	(1.0)	11	(6.8)	
Cardiomyopathy	7	(2.7)	4	(4.0)	3	(1.9)	
Aortic dissection	6	(2.3)	1	(1.0)	5	(3.1)	
**SCAI-Classification of CS**, *n* (%)							
Stage A	0	(0.0)	0	(0.0)	0	(0.0)	**0.001**
Stage B	6	(0.0)	0	(0.0)	6	(3.7)	
Stage C	95	(36.1)	0	(0.0)	95	(58.6)	
Stage D	20	(7.6)	0	(0.0)	20	(12.3)	
Stage E	142	(54.0)	101	(100.0)	41	(25.3)	
**Transthoracic echocardiography**							
LVEF > 55%, (*n*, %)	27	(10.2)	8	(7.9)	19	(11.7)	
LVEF 54–41%, (*n*, %)	31	(11.8)	11	(10.9)	20	(12.3)	
LVEF 40–30%, (*n*, %)	61	(23.2)	25	(24.8)	36	(22.2)	0.483
LVEF < 30%, (*n*, %)	126	(47.9)	47	(46.5)	79	(48.8)	
LVEF not documented, (*n*, %)	18	(6.8)	10	(9.9)	8	(4.9)	
VCI, cm (median, (IQR))	1.8	(1.5–2.2)	1.8	(1.5–2.2)	1.9	(1.6–2.2)	0.099
TAPSE, mm (median, (IQR))	15	(11–18)	17	(14–21)	14	(11–18)	**0.015**
**Cardiopulmonary resuscitation**							
OHCA, *n* (%)	101	(38.4)	101	(100.0)	0	(0.0)	**0.001**
IHCA, *n* (%)	41	(15.6)	0	(0.0)	41	(25.3)	
No CPR, *n* (%)	121	(46.0)	0	(0.0)	121	(74.7)	
Shockable rhythm, *n* (%)	72	(27.6)	61	(61.0)	11	(6.8)	**0.001**
Non-shockable rhythm, *n* (%)	70	(26.6)	40	(39.6)	30	(18.5)	
ROSC, min (median, IQR)	15	(10–27)	15	(10–29)	10	(5–23)	**0.028**
**Respiratory status**							
Mechanical ventilation, *n* (%)	151	(57.4)	96	(95.1)	55	(34.8)	**0.001**
Duration of mechanical ventilation, days (median, (IQR))	2	(1–6)	5	(2–9)	1	(0–2)	**0.001**
PaO_2_/FiO_2_ ratio, (median, (IQR))	222	(133–354)	192	(120–331)	252	(142–367)	0.166
PaO_2_, mmHg (median, (IQR))	103	(77–165)	112	(79–201)	98	(76–149)	**0.047**
**Multiple organ support during ICU**							
Dosis norepinephrine on admission, µg/kg/min (median, (IQR))	0.1	(0.0–0.3)	0.2	(0.1–0.4)	0.1	(0.0–0.2)	**0.001**
Levosimendan, *n* (%)	68	(25.9)	22	(21.8)	46	(28.4)	0.234
Mechanical circulatory assist device, *n* (%)	25	(9.5)	13	(12.9)	12	(7.4)	0.142
CRRT, *n* (%)	79	(30.0)	29	(28.7)	50	(30.9)	0.711
**Baseline laboratory values**, (median, (IQR))							
pH	7.29	(7.19–7.37)	7.25	(7.15–7.34)	7.31	(7.23–7.38)	**0.001**
Lactate (mmol/L)	3.3	(1.7–7.2)	3.8	(2.2–8.8)	2.8	(1.6–6.1)	**0.021**
Serum sodium (mmol/L)	138	(136–141)	139	(136–140)	137	(135–141)	0.132
Serum potassium (mmol/L)	4.3	(3.8–4.9)	4.2	(3.5–4.6)	4.4	(4.0–5.0)	**0.004**
Serum creatinine (mg/dL)	1.48	(1.12–2.14)	1.41	(1.18–1.76)	1.53	(1.10–2.85)	**0.037**
Hemoglobin (g/dL)	12.4	(10.3–13.9)	13.2	(11.5–14.4)	11.9	(9.7–13.7)	**0.001**
WBC (10^6^/mL)	14.71	(10.49–19.05)	16.39	(12.31–21.81)	13.31	(9.93–17.85)	**0.001**
Platelets (10^6^/mL)	222	(171–275)	225	(175–276)	215	(165–273)	0.726
INR	1.17	(1.08–1.39)	1.17	(1.08–1,35)	1.18	(1.09–1.41)	0.364
D-dimer (mg/L)	9.78	(2.43–32.00)	24.18	(14.28–32.00)	2.86	(1.23–6.87)	**0.001**
AST (U/L)	130	(43–324)	167	(110–416)	94	(32–265)	**0.005**
ALT (U/L)	77	(32–189)	115	(71–202)	51	(27–182)	**0.001**
Bilirubin (mg/dL)	0.63	(0.43–1.00)	0.61	(0.41–0.79)	0.66	(0.43–1.19)	0.061
Troponin I (µg/L)	0.763	(0.164–6.154)	0.731	(0.201–5.047)	0.780	(0.143–8.849)	0.800
NT-pro BNP (pg/mL)	4387	(729–13,595)	1047	(339–4462)	7944	(2459–15,352)	**0.001**
Procalcitonin (ng/mL)	0.28	(0.11–0.96)	0.23	(0.06–1.22)	0.31	(0.12–0.98)	0.594
CRP (mg/L)	13	(4–43)	4	(4–19)	25	(5–69)	**0.001**
**Primary endpoint**							
All-cause mortality at 30 days, *n* (%)	144	(54.8)	64	(63.4)	80	(49.4)	**0.027**
**Follow up data**, *n* (%)							
ICU time, days (median, (IQR))	4	(2–8)	6	(3–10)	3	(2–6)	**0.001**
Death ICU, *n* (%)	143	(54.4)	64	(63.4)	79	(48.8)	**0.021**

ADHF, acute decompensated heart failure; ALT, alanine aminotransferase; AST, aspartate aminotransferase; CRP, C-reactive Protein; CRRT, continuous renal replacement therapy; CS, cardiogenic shock; DIC, disseminated intravascular coagulation; GFR, glomerular filtration rate; ICU, intensive care unit; IHCA, in-hospital cardiac arrest; INR, international normalized ratio; IQR, interquartile range; LVEF, left ventricular ejection fraction; NT-pro BNP, N-terminal pro-B-type natriuretic peptide; OHCA, out-of-hospital cardiac arrest; TAPSE, tricuspid annular plane systolic excursion; VCI, vena cava inferior; WBC, white blood cells. Level of significance *p* < 0.05. Bold type indicates statistical significance.

**Table 3:** Univariate correlations of lactate with laboratory and clinical parameters in patients with non-OHCA (*n* = 162) and OHCA (*n* = 101) at day 1.

	Non-OHCA		OHCA	
	r	*p* Value	r	*p* Value
Age	0.038	0.630	−0.183	0.067
BMI	0.116	0.147	0.068	0.511
WBC (10^6^/mL)	0.052	0.528	0.087	0.404
Platelet count (10^6^/mL)	−0.174	**0.034**	−0.289	**0.005**
Albumin (g/L)	−0.177	**0.040**	−0.465	**0.001**
Bilirubin (mg/dL)	0.295	**0.003**	0.340	**0.012**
CRP (mg/L)	0.113	0.182	0.203	0.050
Procalcitonin (ng/mL)	0.226	0.091	0.551	**0.018**
cTNI (µg/L)	−0.012	0.898	0.264	**0.010**
NT-pro BNP (pg/mL)	0.145	0.228	0.291	0.112
LVEF	0.168	0.033	0.224	**0.025**
PaO_2_/FiO_2_ ratio	−0.024	0.779	−0.269	**0.011**
Mechanical ventilation days	0.039	0.623	−0.407	**0.001**
Creatinine (mg/dL)	0.294	**0.001**	0.240	**0.019**
SOFA score	0.296	**0.001**	0.283	**0.004**
APACHE II score	0.367	**0.001**	0.101	0.313
MAP (mmHg)	−0.104	0.194	−0.197	0.057
Catecholamines	0.258	**0.001**	0.253	**0.012**
Intensive care days	−0.261	**0.001**	−0.441	**0.001**

APACHE II, acute physiology and chronic health evaluation II; BMI, body mass index; CRP, C-reactive protein; cTNI, cardiac troponin I; LVEF, left ventricular ejection fraction; MAP; mean arterial pressure; NT-pro BNP, N-terminal pro-B-type natriuretic peptide; SOFA, sepsis-related organ failure assessment score; WBC, white blood cells. Level of significance *p* < 0.05. Bold type indicates statistical significance.

**Table 4:** Diagnostic performance of lactate at days 1, 2, 3, 4 and 8 analyzed as area under the curve (95% CI).

	Non-OHCA	OHCA
**Day 1**	0.622 (0.535–0.710); ***p* = 0.007**	0.753 (0.658–0.848); ***p* = 0.001**
**Day 2**	0.758 (0.672–0.843), ***p* = 0.001**	0.731 (0.624–0.839); ***p* = 0.001**
**Day 3**	0.784 (0.679–0.889); ***p* = 0.001**	0.720 (0.601–0.838); ***p* = 0.002**
**Day 4**	0.722 (0.592–0.852); ***p* = 0.004**	0.761 (0.644–0.877); ***p* = 0.001**
**Day 8**	0.479 (0.204–0.754); *p* = 0.872	0.863 (0.739–0.987); ***p* = 0.001**

Level of significance *p* < 0.05. Bold type indicates statistical significance.

**Table 5:** Multivariable Cox regression analyses within the entire study cohort.

Variables	Non-OHCA			OHCA		
	HR	95% CI	*p* Value	HR	95% CI	*p* Value
Age	1.020	0.997–1.044	0.087	1.022	1.000–1.045	0.052
Sex	0.930	0.553–1.565	0.786	1.068	0.585–1.947	0.831
WBC	1.078	1.032–1.125	**0.001**	1.005	0.959–1.053	0.836
Platelets	1.000	0.997–1.003	0.873	0.999	0.995–1.002	0.406
Systolic BP	0.993	0.984–1.002	0.121	0.991	0.983–1.000	**0.047**
Respiratory rate	1.024	0.984–1.065	0.246	1.001	0.955–1.050	0.968
Creatinine	1.028	0.904–1.170	0.670	1.100	0.880–1.377	0.403
Catecholamines	1.390	1.094–1.768	**0.007**	1.006	0.742–1.363	0.972
Lactate	1.070	1.009–1.134	**0.024**	1.151	1.071–1.238	**0.001**

BP, blood pressure; OHCA, out-of-hospital cardiac arrest; WBC, white blood cell count. Level of significance *p* < 0.05.

## Data Availability

Not applicable.
